# Popular Science Department

**Published:** 1883-01

**Authors:** 


### Popular Science Department.

Behavior of the White Corpuscle.—On the 7th of August, 1882, I was examining the blood of a patient, a robust man in fine health. The blood seemed in excellent condition ; the proportions of the elements were all that could be desired. The medical history of the case has nothing to do with this observation and hence is omitted.

In the specimen of blood examined there were a few of those glistening round bodies which are present more or less in all blood that I have examined. The name and functions of these bodies are alike unknown to me and are, I believe, matters of dispute among histologists at present ; but whatever end they may fulfill they have been present in the greatest number in the blood of debilitated patients who were for the most part consumptives ; still, as before remarked, they have been observed in the healthiest blood.

In the blood in question the white corpuscles were unusually large and active, and one in particular attracted my attention by the rapidity of its movements. I saw it glide toward one of these glistening bodies and surround it, insinuate itself underneath it, and, so to speak, get the body upon its (the corpuscle's) back. Carrying this body along it approached another and executed the same maneuver, and so with another and another, until six of these bodies of different sizes were upon the white corpuscle. Then one after another sank into the interior of the white corpuscle and could be seen in it as dark specks, the shade gradually becoming less deep, as if the bodies slowly dissolved away, the smallest first disappearing.

The sixth body did not sink into the corpuscle, but the corpuscle slid from under it and left it resting upon the slide. The corpuscle next retreated a short distance and, extending another part of its substance, again approached the body and nearly surrounded it. Again the corpuscle retreated, and again returning, endeavored apparently to surround the body, but unsuccessfully. These movements of apparent attack and retreat were repeated several times, and at last the corpuscle moved off a distance equal to the diameter of six or seven red corpuscles. I thought then the performance was at an end, but no, the corpuscle suddenly stopped and returned again to the body ; this time it had so rolled over as to present to the body the exact place through which the largest of the other five bodies had passed into its interior; it then extended itself out under the body, so as to raise the latter up from the slide. The body now occupied the position of a ball in a cup and began slowly to sink into the corpuscle. Suddenly, however, it slipped out, rolled off of the corpuscle, and fell upon the slide with a little rebound. The white corpuscle now apparently gave up the attack and moved rapidly away. The time of observation was about three and a half hours. The instrument was a one-quarter inch objective (Tolles), a one-inch eye-piece and an amplifier in the tube.

I do not desire to theorize nor draw inferences on this behavior of the white corpuscle, but simply record the facts as I saw them.—*R. J. Nunn in the Microscope.*

The Steam Street Supply in New York.—There still seems to be trouble in keeping the joints tight under our streets. The screw joints do not seem to hold their own, either from inadequate material to give strength to the fittings, unusual strain by expansion, or unskilled labor in screwing the threads home, as fresh outbreaks are of almost daily occurrence.

Screw fittings should be made unusually strong and suited in every particular to the magnitude of the work, for there is no economy, and at most a mere make-shift, in the resort to the use of clamps and putty. The cause that disturbs or ruptures the joint at first will soon affect the clamps.

In our comments upon the progress of the steam supply in our issue of December 9, we aimed to criticise the want of care and time in making up the rubber combination joints. We were far from intending to find fault with the rubber combination itself as a packing

(the Jenkins), which is now so extensively used for steam and other purposes, and has the highest reputation for excellence. We have in mind an example where this packing is now in use with steam under a pressure of from 150 to 225 pounds to the square inch, and was tested to near 300 pounds.

The first screwing up of bolts upon the flanged joints was not final, but gradual, as the heat and pressure was increased, until a solid vulcanite was obtained between the faces of the flanges.—*Scientific American.*

